# TGF-β1 blockade of microglial chemotaxis toward Aβ aggregates involves SMAD signaling and down-regulation of CCL5

**DOI:** 10.1186/1742-2094-7-28

**Published:** 2010-04-29

**Authors:** Wei-Chao Huang, Feng-Chang Yen, Feng-Shiun Shie, Chih-Ming Pan, Young-Ji Shiao, Cheng-Ning Yang, Fong-Lee Huang, Yen-Jen Sung, Huey-Jen Tsay

**Affiliations:** 1 Institute of Neuroscience, Brain Research Center, National Yang-Ming University, Taipei 11221, Taiwan; 2 Division of Psychiatry, Cheng-Hsin Rehabilitation Medical Center, Taipei 11221, Taiwan; 3 Division of Mental Health and Substance Abuse, National Health Research Institute, Zhunan 35053, Taiwan; 4 National Research Institute of Chinese Medicine, Taipei 11221, Taiwan; 5 Institute of Anatomy and Cell Biology, National Yang-Ming University, Taipei 11221, Taiwan

## Abstract

**Background:**

Overactivated microglia that cluster at neuritic plaques constantly release neurotoxins, which actively contribute to progressive neurodegeneration in Alzheimer's disease (AD). Therefore, attenuating microglial clustering can reduce focal neuroinflammation at neuritic plaques. Previously, we identified CCL5 and CCL2 as prominent chemokines that mediate the chemotaxis of microglia toward beta-amyloid (Aβ)aggregates. Although transforming growth factor-β1 (TGF-β1) has been shown to down-regulate the expression of chemokines in activated microglia, whether TGF-β1 can reduce the chemotaxis of microglia toward neuritic plaques in AD remains unclear.

**Methods:**

In the present study, we investigated the effects of TGF-β1 on Aβ-induced chemotactic migration of BV-2 microglia using time-lapse recording, transwell assay, real-time PCR, ELISA, and western blotting.

**Results:**

The cell tracing results suggest that the morphological characteristics and migratory patterns of BV-2 microglia resemble those of microglia in slice cultures. Using this model system, we discovered that TGF-β1 reduces Aβ-induced BV-2 microglial clustering in a dose-dependent manner. Chemotactic migration of these microglial cells toward Aβ aggregates was significantly attenuated by TGF-β1. However, these microglia remained actively moving without any reduction in migration speed. Pharmacological blockade of TGF-β1 receptor I (ALK5) by SB431542 treatment reduced the inhibitory effects of TGF-β1 on Aβ-induced BV-2 microglial clustering, while preventing TGF-β1-mediated cellular events, including SMAD2 phosphorylation and CCL5 down-regulation.

**Conclusions:**

Our results suggest that TGF-β1 reduces Aβ-induced microglial chemotaxis via the SMAD2 pathway. The down-regulation of CCL5 by TGF-β1 at least partially contributes to the clustering of microglia at Aβ aggregates. The attenuating effects of SB431542 upon TGF-β1-suppressed microglial clustering may be mediated by restoration of CCL5 to normal levels. TGF-β1 may ameliorate microglia-mediated neuroinflammation in AD by preventing activated microglial clustering at neuritic plaques.

## Background

Alzheimer's disease (AD) is associated with chronic inflammation induced by overactivated microglia in the central nervous system [1,2]. Overactivated microglia surrounding neuritic plaques persistently release pro-inflammatory mediators and reactive oxygen species, thereby causing a vicious cycle of neuroinflammation and neuronal degeneration [2-6]. The phagocytic role of microglia that cluster around neuritic plaques remains controversial because only minimal amounts of Aβ are internalized by these cells in AD brains [7,8]. It is plausible that preventing the clustering of microglia at neuritic plaques may reduce microglial overactivation and neurodegeneration.

Elevated levels of multiple chemokines and their receptors in AD brains suggest that there is chemotactic migration of microglia toward Aβ plaques [9,10]. For example, the accumulation of microglia is enhanced by the overexpression of CCL2 in transgenic mice overexpressing human amyloid precursor protein (hAPP) [11]; conversely, knockout of the CCL2 receptor diminishes microglial accumulation in these mice [12]. In a prior study, we identified CCL5 as a prominent chemokine that mediates Aβ-induced clustering of BV-2 microglia [13]. These results suggest that chemokines actively participate in microglial chemotaxis toward neuritic plaques and that elevated chemokine levels can recruit more microglia toward these plaques.

Transforming growth factor-β1 (TGF-β1) has multiple roles including modulating inflammation and neuronal survival [14,15]. Upon binding of TGF-β1, type I and type II TGF-β1 receptors activate SMAD2/3 [15]. TGF-β1 signaling has been shown to play a pivotal role in down-regulating chemokines and chemokine receptors [16,17]. In addition, down-regulation of TGF-β type II receptors in neurons has been shown to promote the pathogenesis of AD [14]. The regulatory role of TGF-β1 in AD pathogenesis, therefore, has been intensively examined by over-expressing TGF-β1 or blocking the TGF-β-SMAD signaling pathway [14,18,19] Wyss-Coray *et al*. showed that overexpression of TGF-β1 prominently reduces plaque formation and Aβ accumulation in hAPP mice [19]. Their data also suggest that TGF-β1 enhances Aβ clearance by BV-2 microglia, and, thus, the TGF-β1 pathway may serve as a potential therapeutic target for AD [20]. In fact, a perplexing result in the Wyss-Coray *et al*. report was that, despite the reduction in plaque burden, activated microglia did not appear to cluster around the plaques. Therefore, the role of TGF-β1-SMAD signaling in AD pathogenesis warrants further investigation.

The purpose of this study was to explore the role of TGF-β1 in the chemotaxis of microglia toward Aβ using a cellular approach. Our data suggested that TGF-β1 prevents clustering of microglial cells around Aβ aggregates by attenuating their migration toward neuritic plaques through activation of the SMAD pathway and down-regulation of CCL5.

## Methods

### Preparation of aggregated and fibrillar β-amyloid

Aggregated Aβ25-35 was prepared at 4°C for 60 h and were incubated at 37°C for 48 h [21]. Fibrillar Aβ1-42 and FAM-Aβ1-42 were prepared as described [22]. Briefly, Aβ peptides were dissolved in hexafluoroisopropanol (Sigma-Aldrich, St. Louis, MO) to a concentration of 1 mM and aliquoted. After hexafluoroisopropanol was removed, an Aβ aliquot was suspended in 10 mM HCl at a concentration of 100 μM for 24 h at 37°C to form fibrillar Aβ.

### BV-2 microglia culture

BV-2 cells were maintained in a humidified incubator with 5% CO_2 _at 37°C. The culture medium was Dulbecco's modified Eagle medium (DMEM, Gibco, Grand Island, NY) supplemented with 5% low-endotoxin fetal bovine serum (FBS, HyClone, Logan, UT), 100 units/ml penicillin (Sigma-Aldrich), 100 μg/ml streptomycin (Gibco), and 2 mM L-glutamine (Sigma-Aldrich).

### Primary microglial cultures

Primary rat microglia was prepared as described [23]. Briefly, cortices of neonates at postnatal day one were dissociated by papain and endonuclease followed by trituration. Mixed glial cultures were grown in low-endotoxin medium with 10% fetal bovine serum after 24 h. After 14 days in vitro, microglia were separated from astrocytes by gentle agitation.

### Quantification of microglial clusters

BV-2 cells (5 × 10^4^) were incubated with 3 μM fibrillar Aβ1-42 or 10 μM aggregated Aβ25-35 for 24 h. TGF-β1 pretreatment was carried out 1 h before the addition of Aβ. The distribution of clustered BV-2 cells was photographed as described [13]. A cluster of BV-2 cells was defined as more than 10 cells stacked upon each other surrounding aggregated Aβ. The experiments were repeated at least three times.

### Transwell assay

BV-2 cells and rat primary microglia (3.5 × 10^4^) were seeded in the inserts of transwells (Corning Costar Corp., Cambridge, MA, USA, 8.0 μm pore size) with or without a TGF-β1 pretreatment for 30 min. The transwell assay was performed as described [24]. The insert was transferred into a well containing serum-free DMEM with or without Aβ in the lower compartment and incubated for 2 h in 5% CO_2 _at 37°C. Microglia that migrated to the lower surface were stained with Hoechst 33258. Images were taken from four random fields with a florescent microscope at 4× magnification. The number of microglia on the lower surface of the insert was quantified. The experiments were repeated at least three times.

### Cell tracing and migration speed analysis

BV-2 cells (2 × 10^5^) were seeded in a 35-mm plate and incubated for 2 h. The migration of BV-2 treated with aggregated FAM-labeled Aβ was recorded by time-lapse recording as described [13]. The migration trace of BV-2 cells was recorded over an area of 400 × 368 μm^2 ^between frames 1 and 105. The coordinates of cell tracings were graphed using Microsoft Excel. More than 10 randomly selected cells from each experiment were tracked for four periods: 0-2.5 h, 5-7.5 h, 10-12.5 h, and 15-17.5 h after the addition of Aβ. The cell track between two frames was processed by the "track point" function of MetaMorph. The migration speed was calculated by dividing the distance traveled by the cell over two frames by 3 min. Cells stuck in the cluster were excluded from speed analysis. Speeds derived from tracking a single cell over 50 frames during a 2.5-h period were averaged. Speed distributions of more than 10 cells from each experiment were analyzed. The migration speeds of cells were ranked into three classes: 0-1.5 μm/min, 1.5-3 μm/min, and greater than 3 μm/min. The average speeds of more than 30 cells were calculated from at least three independent recordings.

### Western blotting analysis

BV-2 cells were starved in serum-free medium for 4 h before treatments. BV-2 cells were treated with TGF-β1 for the indicated durations, and cell lysates were then prepared. The inhibitory effects of SB431542 on TGF-β1-induced SMAD2 phosphorylation were examined by SB431542 pretreatment for 30 min followed by TGF-β1 treatment for 30 min. Briefly, cell lysates collected at the indicated time points were subjected to western blot assay using antibodies against phospho-SMAD2, SMAD 2 (1:500), and monoclonal mouse actin antibody (1:2,000, Sigma-Aldrich) individually, followed by either goat anti-rabbit antibody (1:1,000, Santa Cruz Biotechnology, Santa Cruz, CA, USA) or goat anti-mouse antibody (1:1,000, Upstate, Virginia, USA) for 1 h. The immune complex was detected by Western Lightning Western Blot Chemiluminescence Reagent (PerkinElmer Life Sciences, Boston, MA). The experiments were repeated three times.

### Real-time PCR

Real-time PCR amplification of cDNA prepared from BV-2 cells after different treatments was performed using the SYBR Green qPCR Master Mix method (Fermentas Life Sciences, Burlington, Ontario, Canada) in a Corbett Rotor-Gene 3000 system. The levels of *CCL2 *and *CCL5 *mRNA were quantified. The experiments were repeated at least three times. The primer sequences of each gene were as follows: *GAPDH *sense primer: 5'-CCTTCCGTGTTCCTACCC, antisense primer: 5'-AAGTCGCAGGAGACAACC; *CCL5 *sense primer: 5'-TGCCCACGTCAAGGAGTATTT; antisense primer: 5'-TCTCTGGGTTGGCACACACTT; *CCL2 *sense primer: 5'-TGAATGTGAAGTTGACCCGT; and antisense primer: 5'-AAGGCATCACAGTCCGAGTC.

### Quantification of chemokines by ELISA

Medium from BV-2 cells after different treatments was collected for ELISA. Concentrations of CCL2 and CCL5 in the medium were assayed using a mouse CCL2/JE and CCL5/RANTES DuoSet ELISA Development kit (R&D Systems, Minneapolis, MN, USA) following the manufacturer's instructions and detected by an ELISA reader (Labsystems Multiskan RC, Helsinki, Finland). The experiments were repeated at least three times.

### Statistical analysis

Data was analyzed by one-way analysis of variance (ANOVA), followed by the Tukey's HSD (honestly significant difference) test, using Statistical Analysis System software (SAS, SAS Institute Inc., Cary, NC). A *p *value less than 0.05 was considered statistically significant and marked with an asterisk in the figures.

## Results

### Aβ-induced chemotactic migration of BV-2 microglia is attenuated by TGF-β1

**Figure 1:**
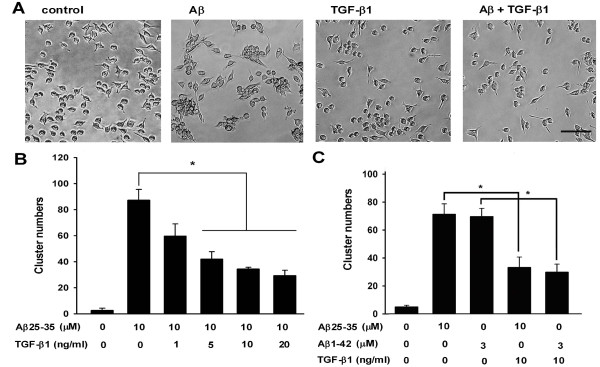
**TGF-β1 attenuated Aβ-induced microglial clustering in a dose-dependent manner**. BV-2 cells were treated with TGF-β1 for 1 h, followed by 10 μM aggregated Aβ25-35 for 24 h. Images of control, TGF-β1-, and Aβ-treated BV-2 cells, with and without TGF-β1 treatment, were taken with a phase-contrast microscope **(A)**. Images were taken from four random fields with a phase-contrast microscope to quantify the number of microglial clusters (B). BV-2 cells were treated with TGF-β1 at different doses for 1 h, followed by aggregated Aβ25-35 or fibrillar Aβ1-42 for 24 h. The number of BV-2 clusters was counted. Bars represent the mean ± SEM. * = *p *< 0.05. Scale bar: 50 μm.

The chemotactic migration of microglia toward Aβ aggregates has been previously suggested [11-13]. Since TGF-β1 down-regulates the expression of chemokines in microglia, whether TGF-β1 regulates Aβ-induced clustering of BV-2 microglia cells was examined. Our results indicate that TGF-β1 dramatically diminishes clustering of BV-2 microglia at aggregated Aβ25-35 (Fig. 1A). The suppressive effect of TGF-β1 on Aβ-induced microglial clusters was significant at 5, 10, and 20 ng/ml (Fig. 1B). The clusters of BV-2 cells induced by both aggregated Aβ25-35 and fibrillar Aβ1-42 were reduced after TGF-β1 treatment by more than 50% (Fig. 1C).

**Figure 2:**
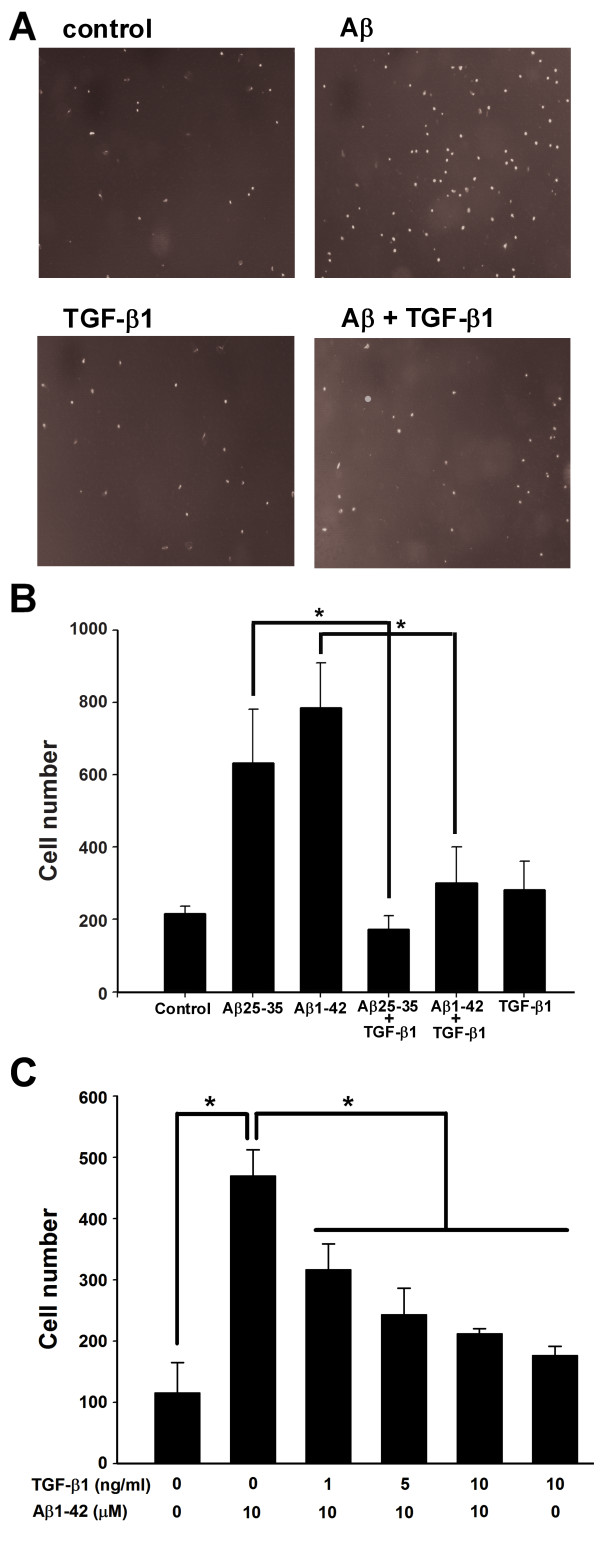
**TGF-β1 suppressed Aβ-induced microglial chemotaxis in the transwell assay**. BV-2 cells were seeded in the inserts of transwells with or without 10 ng/ml TGF-β1. The inserts were transferred to transwells containing 10 μM Aβ25-35 and incubated for 2 h. Images of control, TGF-β1-, and Aβ-treated BV-2 cells, with and without TGF-β1 treatment, were taken from four random fields at 4× magnification **(A)**. The cell number in the lower chamber of the insert was quantified **(B)**. Primary microglia were seeded in inserts of transwells with or without TGF-β1 at different concentrations and incubated for 30 minutes. The cells in the lower chamber of the insert were quantified **(C)**. Bars represent the mean ± SEM. * = *p *< 0.05.

The transwell assay was then used to examine whether TGF-β1 could attenuate the chemotaxis of BV-2 microglia. TGF-β1 (10 ng/ml) effectively reduced the number of BV-2 microglia migrating toward the lower chamber that contained either aggregated Aβ25-35 or fibrillar Aβ1-42 (Fig. 2A, B). Consistently, primary microglia migrated to the lower chamber containing fibrillar Aβ1-42 (Fig. 2C). The suppressive effect of TGF-β1 on the Aβ-induced migration of primary microglia in the transwell assay was significant at 1, 5, and 10 ng/ml.

**Figure 3:**
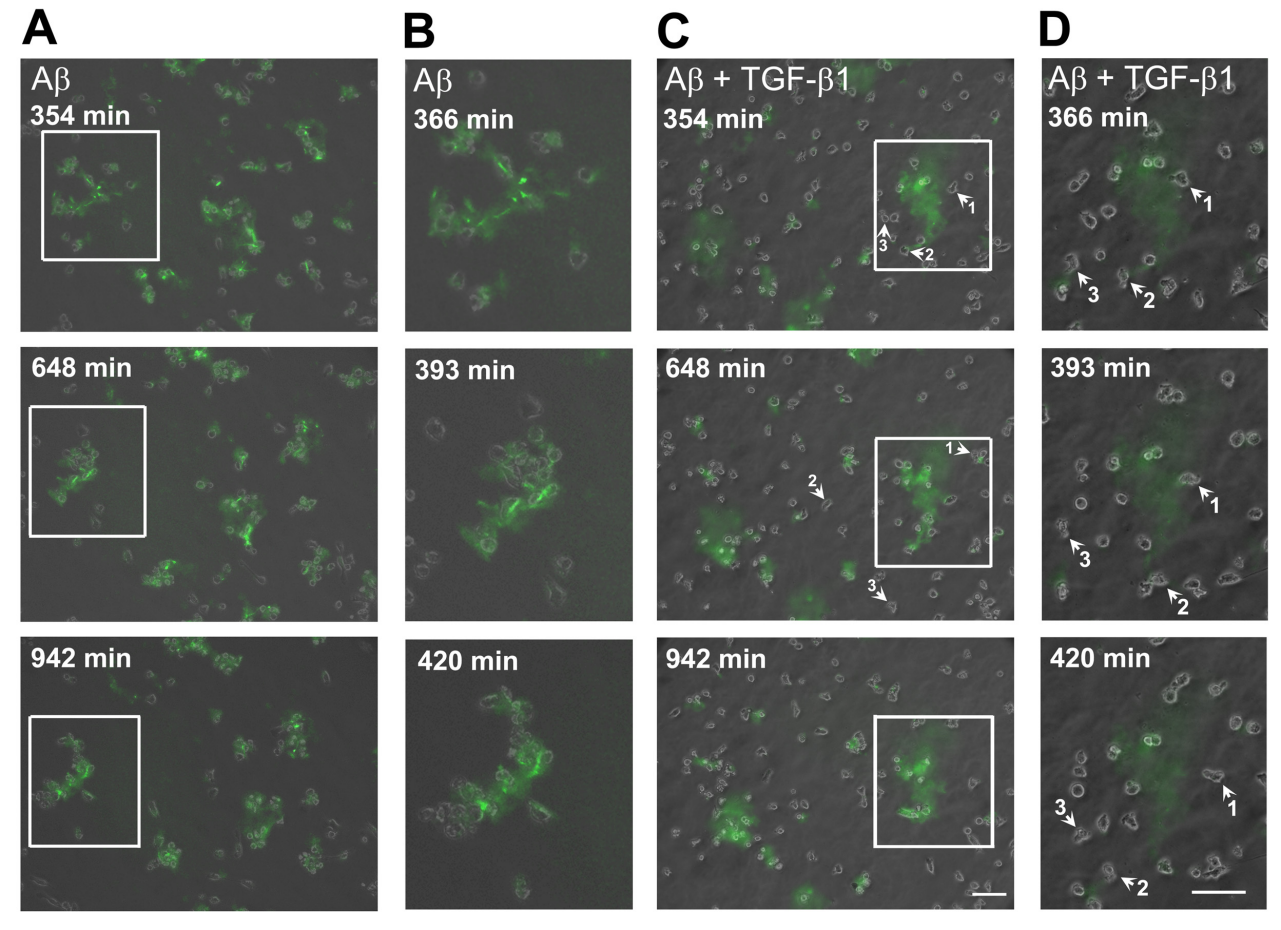
**The differential migratory behavior of Aβ-treated microglia in the absence and presence of TGF-β1**. BV-2 cells were pretreated with 10 ng/ml TGF-β1 for 1 h, followed by FAM-labeled aggregated Aβ. Continuous cellular movements were recorded by time-lapse microscopy from more than three independent experiments. The images (top to bottom) indicate frames recorded at the indicated times, respectively **(A, C)**. BV-2 cells formed clusters at Aβ aggregates between 354 min and 942 min after the addition of Aβ **(A)**. The addition of TGF-β1 attenuated BV-2 clustering at Aβ aggregates **(C)**. Cell movement of the marked areas in A and C was monitored between 366 and 420 min at a higher magnification (**B, D)**. Instead of clustering toward the Aβ aggregates, cells # 1-3 migrated away from Aβ without stalling. Scale bar: 100 μm.

To further characterize the effects of TGF-β1 on the migratory behavior of Aβ-treated microglia, time-lapse recording of BV-2 cells was performed (Fig. 3). Cytoplasmic extensions of BV-2 cells protruded forward in the direction of movement with a long tail. This movement resembled activated microglia in brain slices induced by forebrain stab lesions [25]. The real-time migration of control, TGF-β1-, and Aβ-treated BV-2 cells with and without TGF-β1 is presented as in Additional file 1, Additional file 2, Additional file 3 and Additional file 4. After 354 min, the addition of aggregated Aβ caused two to three BV-2 cells to cluster. After 648 min, the size of the BV-2 clusters increased (Fig. 3A, B). In contrast, TGF-β1 attenuated the formation of Aβ-induced BV-2 clusters (Fig. 3C, D). Figure 3B and 3D show the marked areas in Figure 3A and 3C, illustrating the movement of BV-2 cells at a higher magnification 366 and 420 min following the addition of Aβ. During this time, the clustered BV-2 cells surrounded the aggregated Aβ (Fig. 3B). In the TGF-β1-pretreated group, cells #1-3, located in the vicinity of aggregated Aβ, migrated away from the Aβ aggregates (Fig. 3D). These results suggest that the driving force of chemotaxis toward Aβ aggregates is reduced in the TGF-β1-pretreated group.

**Figure 4:**
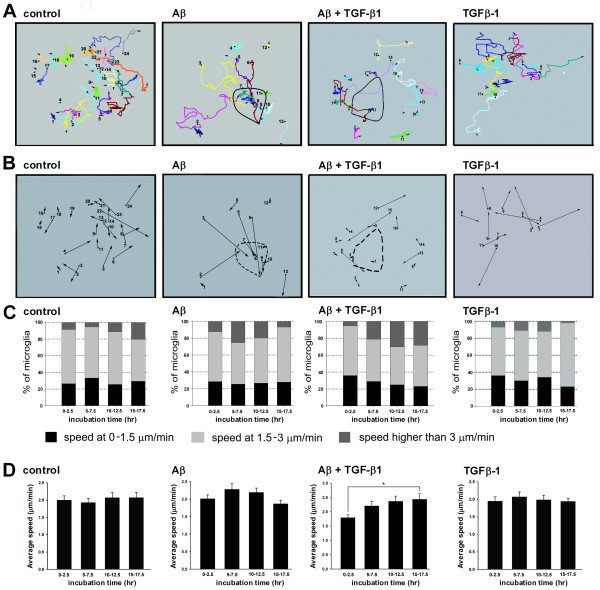
**Cell tracings and speed analysis of Aβ-treated microglia in the absence and presence of TGF-β1**. The migratory tracks of control, TGF-β1-, and Aβ-treated BV-2 cells, with and without TGF-β1 were labeled in different colors **(A)**. The migratory direction of BV-2 cells are represented by the vectors linking the start and end points **(B)**. Speed distributions were analyzed during 0-2.5, 5-7.5, 10-12.5, and 15-17.5 h after the addition of Aβ aggregates **(C)**. Black bars represent the percentage of cells with a speed between 0-1.5 μm/min. Light gray bars represent the percentage of cells with a speed between 1.5-3 μm/min. Dark gray bars represent the percentage of cells with a speed greater than 3 μm/min. Average speeds were calculated by averaging the migration speed during the entire recording period **(D)**. Bars represent the mean ± SEM. Comparisons marked with asterisks are significant different. At least 10 cells from each independent time-lapse recording were pooled for speed distribution and average speed analysis. * = *p *< 0.05.

A representative cell tracing shows that control, TGF-β1-treated, and TGF-β1-pretreated Aβ-treated cells migrate randomly for long distances without any defined direction (Fig. 4A). However, Aβ-treated BV-2 cells migrated toward Aβ aggregates. Six hours after the addition of Aβ, more than 50% of traced microglia clustered at Aβ aggregates (Fig. 4B). Once these cells formed clusters, they rarely migrated away from the Aβ aggregates. In the TGF-β1-pretreated Aβ-treated group, less than 14% of traced microglia migrated toward the Aβ aggregates. Conversely, TGF-β1-pretreated cells encountered Aβ aggregates, and then subsequently migrated away from these aggregates. These data support the conclusions drawn from the transwell assay that TGF-β1 attenuates the chemotaxis of microglia toward Aβ.

To address whether TGF-β1 reduces the migration speed of microglia, the speeds of four groups were analyzed (Fig. 4C). The speeds of BV-2 microglia greatly decreased when interacting with other cells, which also resembled those of activated microglia induced by forebrain stab lesions [25]. More than 50% of both control and TGF-β1-only cells migrated between 1.5-3 μm/min. However, the number of cells migrating at speeds greater than 3 μm/min increased, while the number of cells migrating at speeds between 0-1.5 μm/min decreased in the Aβ-treated group. The average migration speed of microglia in the control and TGF-β1 groups was approximately 2 μm/min. The average speed of the Aβ-treated group did not change with time. Surprisingly, the migration speeds of the TGF-β1-pretreated Aβ-treated group increased significantly between 15-17.5 h following the addition of Aβ. Therefore, we may conclude that the attenuation of BV-2 clustering at Aβ by TGF-β1 was not a result of reduction in migration speed of microglia.

### TGF-β1 type I receptor mediates the suppressive effects of TGF-β1 on Aβ-induced microglia clustering

**Figure 5:**
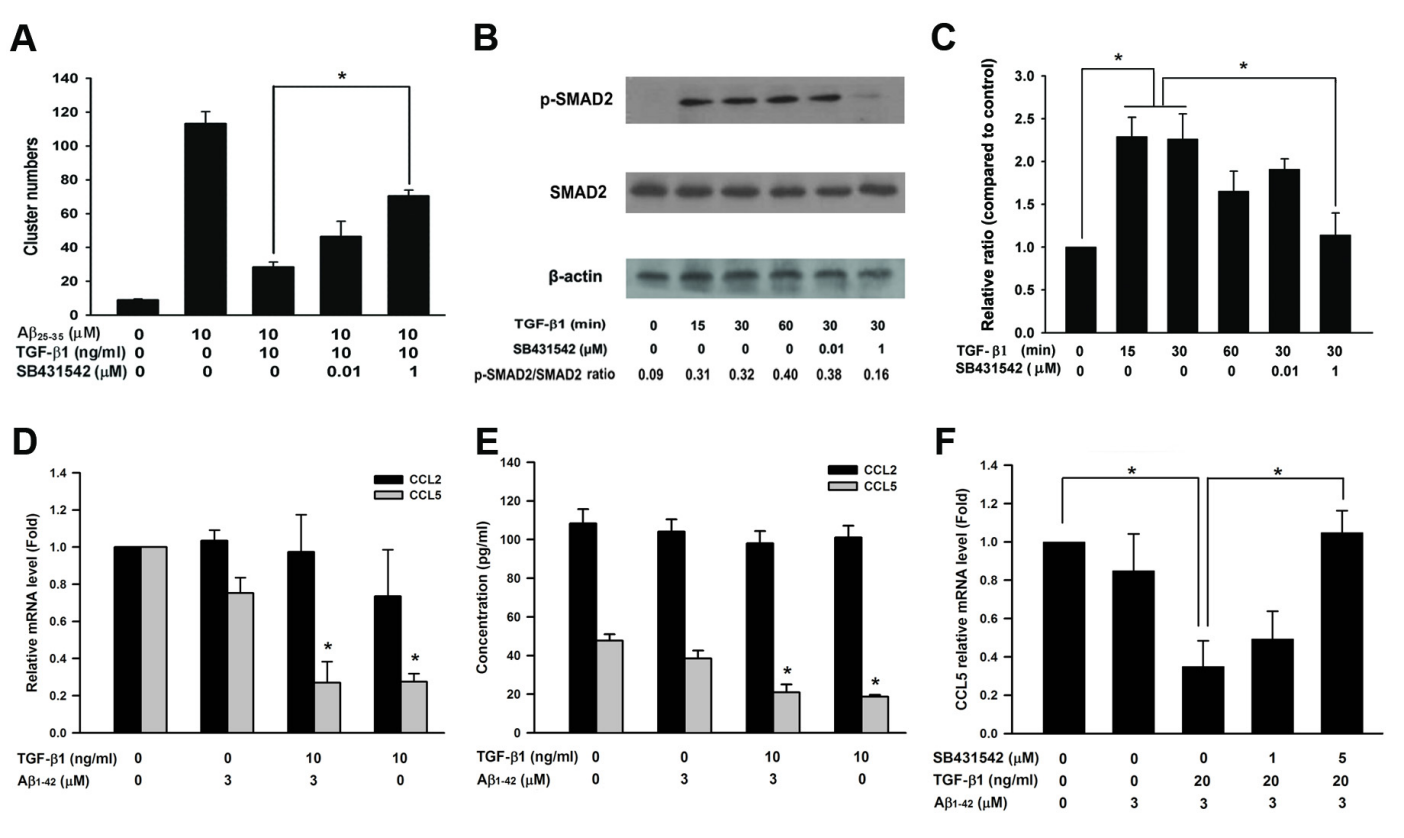
**Phosphorylation of SMAD2 and down-regulation of CCL5 are involved in the attenuation of Aβ-induced microglial clustering by TGF-β1**. BV-2 cells were treated with SB431542 for 1 h prior to the addition of TGF-β1. Subsequently, BV-2 cells were incubated with Aβ. The number of clusters was quantified **(A)**. Representative western blot analysis of phospho-SMAD2 after the addition of TGF-β1 for different durations and the inhibitory effect of SB431542 on SMAD2 phosphorylation (**B**). The relative intensities of phospho-SMAD2 compared to control were analyzed (**C**). The levels of CCL2 and CCL5 mRNA in BV-2 cells after different treatments were compared by real-time PCR **(D)**. ELISA kits were used to measure the levels of CCL2 and CCL5 in the medium of BV-2 cells after different treatments **(E)**. The effects of SB431542 on the down-regulation of CCL5 mRNA levels after TGF-β1 treatment were analyzed **(F)**. Bars represent the mean ± SEM. All of the data were collected from three independent experiments. * = *p *< 0.05.

TGF-β1-SMAD2/3 signaling has been shown to mediate down-regulation of cytokines and chemokines [26,27]. SB431542, an inhibitor of the TGF-β1 type I receptor (ALK5), is commonly used to block SMAD2/3 phosphorylation induced by TGF-β1 [28-31]. To examine whether the TGF-β1 type I receptor mediates the inhibitory effects of TGF-β1 on Aβ-induced microglial cluster formation, SB431542 was used in the assay of microglial clustering. The inhibitory effect of TGF-β1 on Aβ-induced microglial clustering was significantly attenuated by SB431542 (Fig. 5A). Concurrently, SB431542 effectively suppressed TGF-β1-induced SMAD2 phosphorylation in BV-2 microglia (Fig. 5B). The relative intensities of phospho-SMAD2/SMAD2/3 suggest that SB431542 effectively suppressed TGF-β1-induced SMAD2 phosphorylation in BV-2 microglia. Consistent results were observed by using actin and total SMAD2/3 to normalize levels of phospho-SMAD2. Quantitative analysis of western blots of triplicate experiments showed that the level of SMAD2 phosphorylation induced by TGF-β1 was significantly down-regulated by SB431542 (Fig. 5C). Our data suggest that inhibition of SMAD2 phosphorylation, which is a downstream effector of ALK5, can mediate the inhibitory effects of TGF-β1 on Aβ-induced microglia clustering.

Our previous study indicated that both CCL5 and CCL2 mediate the chemotactic migration of microglia toward Aβ aggregates [13]. Whether TGF-β1 down-regulates CCL5 and CCL2 was then investigated. Levels of both CCL5 mRNA and secreted CCL5 in the medium were significantly decreased by TGF-β1 pretreatment in the Aβ-treated group (Fig. 5D, E). However, there were no significant changes in the levels of CCL2 mRNA and secreted CCL2 in the medium of the TGF-β1 pretreated Aβ-treated group. Furthermore, blockade of ALK5 by SB431542 completely reversed the down-regulation of CCL5 mRNA in the TGF-β1 pretreated Aβ-treated group (Fig. 5F). Our data suggest that binding of TGF-β1 activates ALK5 and leads to phosphorylation of SMAD2. Phosphorylated SMAD2 may down-regulate CCL5 and attenuate chemotaxis of microglia toward Aβ aggregates.

## Discussion

It has been suggested that overactivated microglia surrounding neuritic plaques produce proinflammatory cytokines and chemokines that recruit more neurotoxic microglia and lead to further neuroinflammation [1,3-6]. Therefore, attenuation of overactivated microglia at neuritic plaques may be a therapeutic strategy for AD. In the present study, we used a time-lapse recording approach to examine the effects of TGF-β1 on Aβ-induced microglial clustering. Our data suggests that TGF-β1 significantly inhibits microglial chemotaxis toward Aβ. Phosphorylated SMAD2 and down-regulation of CCL5 were at least partially involved in mediating the effects of TGF-β1. These results suggest that TGF-β1 may have therapeutic potential for AD by preventing excessive microglial clustering at neuritic plaques, since microglial overactivation at neuritic plaques actively contributes to neurodegeneration [32].

Although microglia have been shown to be recruited and activated by nearby plaques [33], the trajectory of microglial movement *in vivo *has not been examined because of the difficulty in monitoring microglial clustering at neuritic plaques. Our study provides, for the first time, real-time tracing data of microglial migration toward Aβ aggregates. BV-2 cells have been widely used to study microglial migration induced by alpha-synuclein and cannabinoid [34,35]. The migratory behavior of BV-2 cells is similar to that of primary microglia [36]. Furthermore, the morphology and behavior of migrating BV-2 cells in our study resembled that of microglia in brain slices [25]. Although the clustering of BV-2 microglia around Aβ aggregates *in vitro *may not accurately mimic microglia around neuritic plaques in AD, our approach provides a tractable system for the study of molecular mechanisms of TGF-β1 attenuation of microglia chemotaxis toward neuritic plaques.

TGF-β1 overexpression has been shown to prominently reduce plaque formation and Aβ accumulation in hAPP/TGF-β1 double transgenic mice [19]. It is suggested that TGF-β1 enhances the uptake of Aβ by microglia. However, Town *et al*. showed that the blockade of TGF-β1-SMAD2/3 signaling in peripheral macrophages reduces Aβ deposits in hAPP mice, and they demonstrated that such blockade enhances infiltration of macrophages into the central nervous system [18]. Furthermore, it has been shown that TGF-β1 knockout mice develop spontaneous neurodegeneration [37,38]. Blockade of neuronal TGF-β1 signaling has been shown to reduce neuronal survival rate and enhance amyloid deposition in AD mice [14]. These studies suggest that the function of the TGF-β1-SMAD2/3 pathway is cell-type specific. Therefore, the neuroprotective effects of TGF-β1 in AD may directly promote neuronal survival as well as enhance Aβ uptake and prevent the clustering of overactivated microglia at senile plaques.

Phosphorylation of SMAD2/3, the downstream effector of ALK4, 5, and 7, has been shown to be responsible for the transmission of TGF-β signaling in mammalian cells [15]. Several studies have suggested that SMAD2/3 signaling mediates the down-regulation of chemokines during inflammatory responses [26,27]. SB431542, a specific ALK5 inhibitor, is commonly used to inhibit SMAD2/3 phosphorylation [39-41], and has been used to attenuate the neuronal protective effects of TGF-β1 in Aβ-injected rats [42]. A key finding in this study is the inhibition of SB431542 on TGF-β1-suppressed microglia clustering and its concurrent effects on inhibiting SMAD2 phosphorylation and restoring CCL5 expression. Based on the specificity of SB431542 for the TGF-β1-SMAD2/3 pathway [39-41], a logical conclusion is that TGF-β1 reduces microglial clustering and CCL5 expression by activating the ALK5-SMAD2 pathway. Due to the lack of a SMAD2-specific inhibitor, it is impossible to exclude SMAD3 or other effectors as responsible for the inhibitory effects on microglial chemotaxis toward Aβ. CCL5 can serve as a microglia activator and a chemokine in dendritic cells [43]. Moreover, the deletion of the *CCL5 *gene significantly reduces glial activation induced by different stimuli, suggesting that CCL5 can activate microglia [44]. Thus, down-regulation of CCL5 by TGF-β1 at neuritic plaques may prevent sequential microglial clustering and microglia activation.

Although numerous studies suggest that microglia surrounding senile plaques express pro-inflammatory cytokines, Colton *et al*. has shown that genes involved in tissue repair are upregulated in microglia of AD patients and AD mice [45]. Microglia can be beneficial as well as deleterious due to the various phenotypes of microglia under different microenvironments in CNS [46]. For example, the ligation of CD40-CD40L induces TNF-α production. This proinflammatory phenotype is attenuated by TGF-β1 and IL-10 [47]. Different types of stimuli will lead to distinct microglial responses; therefore, the broad spectrum of microglial activation should be evaluated during the development of therapeutic strategies of AD.

## Conclusions

Taken together, our results suggest that TGF-β1 reduces chemotaxis of microglia toward Aβ aggregates, and that this effect at least partially involves down-regulation of CCL5. CCL5 down-regulation via ALK5-SMAD2 signaling may reduce neuroinflammation and neuronal damage in AD. TGF-β1 should be further explored as a potential target for the development of therapeutic strategies to treat AD.

## Competing interests

The authors declare that they have no competing interests.

## Authors' contributions

WCH and FCY performed real-time recording and quantified the number of microglia clusters. FSS and CMP performed real-time PCR and ELISA. CNY and FLH performed transwell assays and western blotting. YJS and HJT were involved in the experimental design and wrote the manuscript. All authors have read and approved the final version of the manuscript.

## Supplementary Material

Additional file 1 Time-lapse recording of migratory motion of control BV-2 microglia. Click here for file

Additional file 2 Time-lapse recording of migratory motion of BV-2 microglia treated with aggregated FAM-labeled Aβ. Click here for file

Additional file 3 Time-lapse recording of migratory motion of BV-2 microglia treated with TGF-β1. Click here for file

Additional file 4 Time-lapse recording of migratory motion of BV-2 microglia treated with TGF-β1 and aggregated FAM-labeled Aβ. Click here for file
